# Perinatal factors, growth and development, and osteosarcoma risk

**DOI:** 10.1038/sj.bjc.6603474

**Published:** 2006-11-14

**Authors:** R Troisi, M N Masters, K Joshipura, C Douglass, B F Cole, R N Hoover, and the National Osteosarcoma Etiology Group

**Affiliations:** 1 Division of Cancer Epidemiology and Genetics, National Cancer Institute, National Institutes of Health, Department of Health and Human Services, Bethesda, MD, USA; 2 Department of Community and Family Medicine, Dartmouth Medical School, Hanover, NH, USA; 3 Westat, Rockville, MD, USA; 4 Department of Oral Health Policy and Epidemiology, Harvard School of Dental Medicine, Boston, MA, USA; 5 Department of Epidemiology, Harvard School of Public Health, Boston, MA, USA; 6 University of Puerto Rico Medical Sciences Campus, San Juan, Puerto Rico

**Keywords:** osteosarcoma, birth weight, height, growth, puberty

## Abstract

Osteosarcoma incidence patterns suggest an aetiologic role for perinatal factors, and growth and development. Osteosarcoma patients (*n*=158) and controls with benign orthopaedic conditions (*n*=141) under age 40 were recruited from US orthopaedic surgery departments. Exposures were ascertained by interview, birth, and growth records. Age- and sex-adjusted odds ratios (OR) and 95% confidence intervals (CI) were estimated. Current height and age- and sex-specific height percentiles were not associated with osteosarcoma risk. Male cases, however, appeared to have an earlier adolescent growth period, and earlier attainment of final height (OR=7.1; 95% CI=1.6–50 for <19 *vs* 19+ years), whereas earlier puberty appeared protective with ORs of 0.41 (95% CI 0.18–0.89) and 0.68 (95% CI 0.31–1.5) for developing facial and pubic hair, respectively. High birth weight was associated with an elevated osteosarcoma risk (OR=3.9; CI=1.7–10 for 4000 g *vs* 3000–3500 g), although there was no trend in risk with increasing weight. These data provide some evidence that osteosarcoma is related to size at birth and in early adolescence, while earlier puberty in male subjects may be protective.

There are few established osteosarcoma risk factors apart from early exposure to high-dose radiation, and Paget's disease, hereditary retinoblastoma, and Li–Fraumeni Syndrome (Miller *et al*, 1996). The bimodal age–incidence curve reflecting peak rates occurring both in adolescence and in older age suggests two separate aetiologies. Enhanced carcinogenic susceptibility during the adolescent growth period is suggested by higher radiogenic bone cancer risk among children than adults, and the characteristic development of childhood tumours in the long bone epiphyses of the lower limbs (Fraumeni, 1967). An excess osteosarcoma risk in larger compared with smaller dog breeds may be consistent with this hypothesis (Withrow *et al*, 1991). Higher male than female incidence rates in puberty, and the early age at which osteosarcoma incidence first peaks – 10–14 years of age in girls, and 15–19 years in boys – may indicate the importance of accelerated growth and hormonal differences, and raise the possibility that very early-life exposures play a role as well (SEER, 2005).

In this study, we focused on the associations of osteosarcoma with factors related to growth and development from the *in utero* period through puberty and adolescence.

## MATERIALS AND METHODS

### Study population

Participants were drawn from orthopaedic surgery departments in 10 US medical centres between 1994 and 2000 (Massachusetts General Hospital, Boston, MA, USA; Creighton University/St Joseph's Hospital and University of Nebraska, Omaha, NE, USA; Children's National Medical Center and Washington Hospital Center, Washington, DC, USA; University of Chicago and Rush Presbyterian St Luke's, Chicago, IL, USA; University of Florida, Gainesville, FL, USA; University of California, Los Angeles, CA, USA; Cleveland Clinic, Cleveland OH, USA). Cases were patients with newly diagnosed primary osteosarcoma admitted for evaluation of eligibility for limb salvage surgery. Controls were orthopaedic patients from the same departments with benign tumours (26%), or non-neoplastic conditions such as inflammatory diseases, cysts, and trauma (ICD-9-CM codes 289.1, 277.8, 354.0, V711, 682.6, 714.3–756.59, 810.0–885.0, and 959.7). Nurse coordinators were responsible for control selection by identifying the next patient matching the case on age (+/−5 years), sex, hospital, and distance from the respective medical centre based on the participant's residential zip code. Pathology reports from surgery and/or biopsy were obtained for all cases and for controls for whom this documentation was applicable to confirm diagnosis, whereas hospital medical records confirmed diagnoses for controls without a pathology report (with conditions such as injury, reconstruction, revision, or pain).

Institutional review boards at each of the medical centres approved this study, and informed consent, which included a check list for a questionnaire, blood draw, and toenail collection, was obtained from all participants. Of eligible cases, 13.6% were not enrolled and a further 3% declined to participate in any of the study components. Of those enrolled in the study, 93.5% of cases and 98% of controls completed interviews. Reasons for non-participation in the questionnaire component included refusal, death, extreme illness following surgery and failure to return for follow-up care. The present analysis was restricted to 169 cases and 144 controls less than 40 years of age to focus on the aetiology of the adolescent-young adult peak in osteosarcoma incidence.

### Exposure and covariate information

Interviews ascertaining information on growth and development, physical activity, and medical history were conducted in the hospital, clinic, or ward with cases after surgery, and after surgery, or other therapy in controls. A supplemental interview on pregnancy exposures was conducted with parents of participants less than 20 years of age, whereas participants over 20 were asked directly.

Participants 9–21 years of age were asked to give consent for growth records acquisition. Of 109 cases and 91 controls, respectively, all of the 78 (71.5%) and 57 (62.6%) participants who reported a regular health-care provider gave permission for contact, and a total of 402 growth records were obtained for 49 cases and 46 controls (average of 4.2 records per participant). Growth records validated participants' self-reports of height (shorter, about the same, and taller) compared with their peers at various ages. Among the combined cases and controls, mean height from records was 9% lower and 1.5% higher among participants who reported being shorter and taller, respectively, compared with being about the same height as their peers at age 9 or 10. Similarly, recorded height was 7% lower and 4.5% higher at age 12 or 13, and 3.5% lower and 6% higher at age 15 or 16. Results were similar when evaluated separately for cases and controls.

US born participants were asked for their consent to acquire birth records from state vital records offices with 97% (*n*=154) of cases and 99% (*n*=139) of controls providing permission. For states where at least six participants were born records were obtained for a total of 289 participants. Equal proportions of cases (55%; *n*=87) and controls (55%; *n*=78) were missing information in the birth records for pregnancy length. Pregnancy complications were missing for 54% (*n*=85) of cases and 59% (*n*=83) of controls, and of those with these data, complications were rare (two cases/five controls). Birth weight as reported by the participant or mother (mean=3392; s.d.=589) and from records (mean=3389; s.d.=556) were highly correlated in those with both sources of information (*r*=0.95; *n*=176). Therefore, reported birth weight was used when birth records were unavailable.

### Statistical analysis

Current height for all participants was converted to percentiles based on sex- and age-specific growth standards provided by the National Center for Health Statistics (CDC, 2000). Unconverted height was analysed in participants 21 years of age or older who were assumed to have attained their final height. Tertiles were based on sex-specific distributions in the control group. Birth weight percentiles were based on gestational age and sex (Oken *et al*, 2003) and quartiles were based on the control distribution. Unconditional logistic regression models including age and sex were used to estimate odds ratios (OR) and 95% confidence intervals (CI). Analyses were based on male and female subjects combined to increase the sample size but are presented separately when findings differed by sex. Further inclusion of study centre, geographic ring, education, and family income did not affect the estimates. Linear trends were assessed using orthogonal polynomial contrasts (Winer, 1962) or by including the continuous variable in the model. The data analysis was generated using SAS/STAT software (1999).

## RESULTS

Similar proportions of cases (64%) and controls (62%) were under 20 years of age, and they were comparable in sex and race/ethnicity (Table 1). Cases had a lower combined family income. There were no trends in age- and sex-adjusted osteosarcoma risk with increasing participant's education, or with mother's or father's education (data not shown).

Neither height percentile nor absolute height was associated with osteosarcoma risk (Table 2). In participants 21–39 years of age, attaining final height at a younger age was associated with a reduced risk in female subjects (OR=0.53; CI=0.15–1.8 for <17 *vs* 17+ years), but with an elevated risk in male subjects (OR=7.1 CI=1.6–50 for <19 *vs* 19+) (*P*-value for interaction=0.01). The association of participant's height percentile with risk showed no consistent pattern in any subgrouping according to mother's height (data not shown).

Cases appeared less likely to be shorter than their peers at ages 9–10 and 12–13, and more likely to be taller at ages 15–16, compared with being about the same height (Table 2). The reduced ORs for being shorter at younger ages were consistent in male and female subjects, but the elevated OR for being taller at age 15–16 resulted from an OR of 2.4 in male subjects and 1.0 in female subjects. Among male subjects, controls were more likely than cases to have started shaving, and developing pubic hair early in male controls followed a similar pattern (Table 3). Age at menarche was not associated with risk. Results for the puberty variables were unchanged with adjustment for height percentile (data not shown).

There was no consistent association between sports participation at various ages during childhood and osteosarcoma risk (data not shown). Comparing frequent with less frequent activity (4+/week *vs* <4/week), the OR were generally elevated in female subjects, in particular, at ages 15 or 16 (OR=2.9; CI=1.2–7.4), whereas there was no association in male subjects (data not shown). Excluding controls whose condition on study entry was fracture attenuated the elevated estimate for more frequent activity in female subjects (OR=1.8; CI=0.8–4.6).

Excluding controls whose condition on study entry was a fracture or fibroma, the OR for ever fracturing a bone was 0.68 (CI=0.41–1.1); with further exclusion of fractures occurring within 2 years of questionnaire completion the OR was 0.65. The site of prior fracture was examined to identify whether for any case, the tumour occurred in the same bone; they matched in only two cases (within 1 year and 15 years of diagnosis).

Diagnostic or therapeutic radiation before the present illness was not associated with osteosarcoma risk (OR=1.2; CI=0.75–1.9). Radiation exposure was mainly in the form of routine medical X-rays (88% of cases and 87% of controls).

High birth weight was associated with an increased osteosarcoma risk (Table 4) that was similar when stratified by age <21 *vs* 21+ years (data not shown), but stronger among female subjects (OR=7.2; CI=1.7–50) than male subjects (OR=2.9; CI=1.0–9.1). There was no trend in risk with gestational age (*P*=0.63), and adjustment for gestational age did not change the OR for birth weight. Birth weight percentiles confirmed the increased risk among heavy babies (highest *vs* lowest quartile OR=4.6; 95% CI=1.4–16.4). Longer birth length appeared protective though the association was not linear or statistically significant. The estimates for both birth weight and length were slightly stronger with mutual adjustment (OR=7.6 for birth weight >4000 g *vs* 3000–3500 g; OR for birth length=0.40, 0.32, and 0.34 for 20, 21, and 22+ inches, respectively, *vs* <20 inches). There were no associations between risk and mother's primary job, smoking, and alcohol intake during pregnancy and father's smoking during pregnancy (data not shown).

## DISCUSSION

Osteosarcoma appears to be positively associated with bone growth, based primarily on the rapid rise and fall of incidence rates from adolescence into young adulthood, and buttressed by the typical occurrence of this tumour (approximately 70% (Miller *et al*, 1996)) in the long bone epiphyses and the strong positive association in canines with breed size (Withrow *et al*, 1991), and in particular, height (Ru *et al*, 1998).

Findings for growth and development in human populations, however, are equivocal. There is some evidence (Fraumeni 1967; Gelberg *et al*, 1997; Cotterill *et al*, 2004) of cases being taller than controls, although the differences often derive from inconsistent subgroup findings defined by age, gender, or anatomic site. Other data suggest no differences in height or average growth rate (Operskalski *et al*, 1987; Pui *et al*, 1987; Glasser *et al*, 1991; Buckley *et al*, 1998). Our data show no association with height, but suggest that cases are less likely to be shorter than their peers before and during the early years of adolescent growth. The positive association we observed with birth weight may also be consistent with earlier growth being adverse. The few previous studies of birth size, like those of height, have had conflicting results (Operskalski *et al*, 1987; Gelberg *et al*, 1997; Buckley *et al*, 1998). The increased risk we observed in the lowest birth length category when adjusted for birth weight could be a chance finding given the lack of overall trend, the increased risk with elevated birth weight, and the inconsistent findings from prior studies (Operskalski *et al*, 1987; Gelberg *et al*, 1997). However, our estimate is similar to that of an earlier study (RR for 21.5 *vs* 19.5–20.5 inches=0.59) (Operskalski *et al*, 1987).

The development of secondary sexual characteristics has also been a focus of studies of growth and development in relation to osteosarcoma risk (Gelberg *et al*, 1997; Buckley *et al*, 1998). We found no associations among female subjects, but earlier development of facial and pubic hair appeared protective in male subjects. Like the growth data, previous findings are inconsistent with respect to timing of sexual development (Gelberg *et al*, 1997; Buckley *et al*, 1998).

Together with those of earlier studies, our results may suggest that there is an underlying relationship of growth and maturation patterns with osteosarcoma risk that is likely too weak to explain a meaningful portion of the remarkable age–incidence curve. The shape of this curve, instead of resulting from growth characteristics, may simply be a weakly correlated marker of the true aetiologic event. The abrupt rise and decline in incidence would be consistent with exposure occurring at a specific common time before the peak. Because of the young age of cases, and resultant requirement for a short common exposure interval, the period during *in utero* development seems likely. In fact, the age–incidence curve is similar to that of vaginal clear-cell adenocarcinoma, caused by *in utero* diethylstilbestrol exposure. In that malignancy, the teratogenic/carcinogenic error occurs in the foetus, but is not manifest until the normal hormonal development and maturation of the reproductive system following puberty. Similarly, carcinogenic events related to *in utero* bone development may only become manifest during their major development and maturation in adolescence. We saw no evidence of adverse effects of *in utero* exposure to alcohol or tobacco. Given the above discussion of growth patterns, perhaps the most likely intrauterine exposures are those with independent effects on subsequent growth and development. Prime candidates for consideration might be gene variants responsible for foetal bone development, and/or environmental exposures such as nutritional factors or infections.

Another commonly suggested risk factor for osteosarcoma is prior bone trauma, although epidemiologic studies have not found evidence of an association with the exception of one study (Operskalski *et al*, 1987). We attempted to assess this based on histories of prior fractures, and by frequency of sports participation although neither was associated with excess risk, and prior fracture was associated with decreased risk. Excluding conditions for which prior fractures or other trauma might be a risk factor did not alter our results, but residual bias is possible. However, because only one case reported a fracture of the same bone more than a year before the diagnosis of osteosarcoma, if such trauma were a risk factor, it would seem to account for only a very small portion of disease.

This investigation has several strengths, including a high cooperation rate with an in-person interview conducted by trained interviewers, and a control group of orthopaedic patients, minimising the opportunity for selection bias, particularly for early-life exposures. There are several limitations as well. The case series included only those patients commonly treated by orthopaedic surgeons (those with limb tumours), with other sites underrepresented, for example, skull, jaw, rib, and vertebral column. Because the hospitals are academic centres with a high rate of referral patients, the opportunity for referral bias exists. For this reason we stratified the controls to the distribution of cases according to hospital, and residential distance from the hospital. Risk estimates were virtually unchanged with adjustment for these variables, as well as for family income. The use of orthopaedic controls has potential disadvantages as well. If the factors we investigated are associated with risk for orthopaedic conditions as well as osteosarcoma, we might fail to identify differences. To mitigate this possibility we included a wide variety of diagnoses in the control group. Finally, the relatively small number of cases limited statistical power, however, owing to the rarity of this tumour, our study includes more cases than all but one of previously published studies (Cotterill *et al*, 2004). Larger studies, however, are needed, particularly to investigate subgroup risks.

In conclusion, our results together with those of previous studies do not suggest a clear growth and development pattern that could explain the dramatic young-age peak of this malignancy. However, there are enough signals to speculate that growth and development characteristics may be markers of true aetiologic events to which they are weakly correlated. Given the sharpness of the young incidence peak, the most likely timing of these events is *in-utero* development. A more concerted effort to evaluate this aspect of aetiology is warranted.

## Figures and Tables

**Table 1:** Distributions of characteristics for osteosarcoma cases and controls

	**Cases (*n*=158)**	**Controls (*n*=141)**
	**No. (%)^a^**	**No. (%)^a^**
*Age (y)*		
0–9	8 (5.1)	9 (6.4)
10–14	37 (23.4)	21 (14.9)
15–19	56 (35.4)	58 (41.1)
20–24	28 (17.7)	19 (13.5)
25–29	12 (7.6)	19 (13.5)
30–34	10 (6.3)	7 (5.0)
35–39	7 (4.4)	8 (5.7)
		
*Sex*		
Male	85 (53.8)	74 (52.5)
Female	73 (46.2)	67 (47.5)
		
*Race*		
White	130 (82.3)	114 (80.9)
Black	14 (8.9)	15 (10.6)
Other	14 (8.9)	12 (8.5)
		
*Income*		
<$40 000	68 (43.0)	39 (27.7)
$40 000–60 000	31 (19.6)	33 (23.4)
>$60 000	34 (21.5)	57 (40.4)
Missing	25 (15.8)	12 (8.5)
		
*Participant's education*		
Less than H.S.	90 (57.0)	70 (49.7)
H.S. or equivalent	26 (16.5)	24 (17.0)
Some college	26 (16.5)	24 (17.0)
College or post-grad	15 (9.5)	23 (16.3)
Missing	1 (0.6)	0 (0.0)

H.S.=high school; y=year.

a Percentages do not add to 100 because the variable is presented for a subgroup of cases and controls or because of rounding.

**Table 2:** Age- and sex-adjusted ORs and 95% CIs for participant's and parent's height and osteosarcoma risk

	**Cases (*n*=158)**	**Controls (*n*=141)**	**OR^a^** **95% CI^a^**
	**No. (%)^b^**	**No. (%)^b^**	
*Current height (percentile)*			
<51	57 (36.1)	47 (33.3)	1.0
51–81	43 (27.2)	45 (31.9)	0.78 (0.44–1.4)
>81	56 (35.4)	47 (33.3)	0.95 (0.54–1.7)
			
*Current height (tertiles)* c			
Lowest	14 (8.9)	15 (10.6)	1.0
Middle	16 (10.1)	16 (11.3)	1.1 (0.40–3.0)
Highest	17 (10.8)	18 (12.8)	1.0 (0.38–2.7)
			
*Height comparison to peers at age 9 or 10* d			
Shorter	27 (17.1)	36 (25.5)	0.56 (0.30–1.0)
About the same	74 (46.8)	57 (40.4)	1.0
Taller	52 (32.9)	41 (29.1)	0.98 (0.57–1.7)
			
*Height comparison to peers at age 12 or 13* e			
Shorter	27 (17.1)	33 (23.4)	0.66 (0.35–1.2)
About the same	64 (40.5)	52 (36.9)	1.0
Taller	47 (29.7)	43 (30.5)	0.88 (0.51–1.5)
			
*Height comparison to peers at age 15 or 16* f			
Shorter	24 (15.2)	26 (18.4)	1.0 (0.53–2.0)
About the same	52 (32.9)	58 (41.1)	1.0
Taller	36 (22.8)	25 (17.7)	1.6 (0.86–3.1)
			
*Mother's height (cm)*			
<162.0	57 (36.1)	36 (25.5)	1.0
162.0–167.6	66 (41.8)	67 (47.5)	0.59 (0.34–1.0)
>167.6	31 (19.6)	38 (27.0)	0.49 (0.26–0.92)
			
*Father's height (cm)*			
<175.3	62 (39.2)	48 (34.0)	1.0
175.3–180.3	34 (21.5)	43 (30.5)	0.63 (0.35–1.1)
>180.3	56 (35.4)	47 (33.3)	0.89 (0.51–1.5)

a OR=odds ratio; CI=confidence interval.

b Percentages do not add to 100 because of either missing data or the variable is presented for a subgroup of cases and controls.

c Among participants 21+ years of age; tertile cutpoints for male subjects are 177.8 and 182.9 cm and for female subjects are 162.6 and 170.2 cm.

d Among participants 9+ years of age.

e Among participants 12+ years of age.

f Among participants 15+ years of age.

**Table 3:** Age- and sex-adjusted ORs and 95% CIs for development measures and osteosarcoma risk

	**Cases (*n*=158)**	**Controls (*n*=141)**	**OR^a^ 95% CI^a^**
	**No. (%)^b^**	**No. (%)^b^**	
*Reached menarche* c			
No	8 (5.1)	8 (5.7)	1.0
Yes	65 (41.1)	59 (41.8)	1.5 (0.46–5.2)
			
*Age at menarche (y)* d			
<12	17 (10.8)	14 (9.9)	1.0 (0.39–2.7)
12	18 (11.4)	16 (11.3)	1.0 (0.40–2.7)
13	19 (12.0)	17 (12.1)	1.0
14+	11 (7.0)	12 (8.5)	0.85 (0.29–2.5)
			
*Started shaving* e			
No	33 (20.9)	16 (11.3)	1.0
Yes	52 (32.9)	53 (37.6)	0.41 (0.18–0.89)
			
*Age first shaved (y)* f			
<15	10 (6.3)	20 (14.2)	1.0
15	21 (13.3)	13 (9.2)	3.2 (1.1–9.1)
16	15 (9.5)	9 (6.4)	3.1 (1.0–10)
17+	6 (3.8)	11 (7.8)	0.99 (0.27–3.5)
			
*Developed pubic hair* e			
No	23 (14.6)	15 (10.6)	1.0
Yes	62 (39.2)	58 (41.1)	0.68 (0.31–1.5)
			
*Age first developed pubic hair (y)* g			
<13	18 (11.4)	22 (15.6)	0.65 (0.28–1.5)
13	28 (17.7)	22 (15.6)	1.0
14+	16 (10.1)	14 (9.9)	0.90 (0.36–2.2)

a OR=odds ratio; CI=confidence interval; y=year.

b Percentages do not add to 100 because of either missing data or the variable is presented for a subgroup of cases and controls.

c Among female subjects.

d Among female subjects who have reached menarche.

e Among male subjects.

f Among male subjects who have started to shave.

g Among male subjects who have developed pubic hair.

**Table 4:** Age- and sex-adjusted ORs and 95% CIs for pregnancy and birth characteristics and osteosarcoma risk

	**Cases (*n*=158)**	**Controls (*n*=141)**	**OR^a^ 95% CI^a^**
	**No. (%)^b^**	**No. (%)^b^**	
*Participant's birth order*			
First	72 (45.6)	63 (44.7)	1.0
Second or later	81 (51.3)	74 (52.5)	0.97 (0.61–1.5)
			
*Gestational age reported by participants*			
Earlier	38 (24.1)	37 (26.2)	0.90 (0.51–1.6)
On time	76 (48.1)	67 (47.5)	1.0
Later	36 (22.8)	30 (21.3)	1.0 (0.58–1.9)
			
*Birth weight (g)*			
<3000	28 (17.7)	27 (19.1)	1.3 (0.66–2.5)
3000–3499	48 (30.4)	58 (41.1)	1.0
3500–3999	41 (25.9)	37 (26.2)	1.3 (0.73–2.4)
4000+	27 (17.1)	8 (5.7)	3.9 (1.7–10)
			
*Birth length (inches)*			
<20	32 (20.3)	16 (11.4)	1.0
20	18 (11.4)	18 (12.8)	0.54 (0.21–1.3)
21	44 (27.8)	45 (31.9)	0.51 (0.24–1.1)
22+	19 (12.0)	12 (8.5)	0.83 (0.31–2.2)

a OR odds ratio; CI confidence interval.

b Percentages do not add to 100 in some cases because of missing data.
